# Role of Oscillations in Auditory Temporal Processing: A General Model for Temporal Processing of Sensory Information in the Brain?

**DOI:** 10.3389/fnins.2018.00793

**Published:** 2018-10-31

**Authors:** Andreas Bahmer, Daya Shankar Gupta

**Affiliations:** ^1^Comprehensive Hearing Center, ENT Clinic, University of Würzburg, Würzburg, Germany; ^2^Biology Department, Camden County College, Gloucester Township, NJ, United States

**Keywords:** canonical microcircuits, cochlear nucleus, locus coerulus, limbic system, amygdala, hippocampus, basal ganglia, substantia nigra

## Abstract

We review the role of oscillations in the brain and in the auditory system showing that the ability of humans to distinguish changes in pitch can be explained as a precise analysis of temporal information in auditory signals by neural oscillations. The connections between auditory brain stem chopper neurons construct neural oscillators, which discharge spikes at various constant intervals that are integer multiples of 0.4 ms, contributing to the temporal processing of auditory cochlear output. This is subsequently spatially mapped in the inferior colliculus. Electrophysiological measurements of auditory chopper neurons in different species show oscillations with periods which are integer multiples of 0.4 ms. The constant intervals of 0.4 ms can be attributed to the smallest synaptic delay between interconnected simulated chopper neurons. We also note the patterns of similarities between microcircuits in the brain stem and other parts of the brain (e.g., the pallidum, reticular formation, locus coeruleus, oculomotor nuclei, limbic system, amygdala, hippocampus, basal ganglia and substantia nigra), dedicated to the processing of temporal information. Similarities in microcircuits across the brain reflect the importance of one of the key mechanisms in the information processing in the brain, namely the temporal coupling of different neural events via coincidence detection.

## 1. Introduction

Oscillations are defined as periodic temporal changes in the state parameters of a system and characterize stable states in the non-linear neural dynamics of the brain. The study of oscillations in the human brain began in the early part of the last century, when neural oscillations were recorded by electroencephalography (EEG) in 1924 by Hans Berger at the University of Jena. Neural oscillations in the EEG recordings are classified according to their frequency in different bands. However, EEG signals are only the summed electrical activity of the brain, as they are measured at the surface of the skull. This averaged activity would mask mechanisms, subserved by oscillations in smaller subpopulations of neurons. Furthermore, invasive single unit recording (extracellular and intracellular) as well as the recording of local field potentials reveal the presence of oscillations.

## 2. The role of oscillations in coupling neural activities in the brain

Neural oscillations are observed in various parts of the brain involving different sensory systems, such as the visual, olfactory, motor, and auditory system. In the midbrain, the presence of neural oscillations in electrophysiological recordings in the auditory system was discovered by Langner (1978), which led to the model of auditory temporal processing and neural oscillators by Langner (1981). Later, neural oscillations became a hot topic of research in the visual system. Studies of Gray and Singer (Gray and Singer, 1989; Gray, 1994), and others (Eckhorn et al., 1988) linked oscillations in the visual system to the binding of various percepts. It was shown that neural oscillations, resulting from the synchronization of spatially segregated retinal ganglion cells evoked by stationary and moving visual stimuli, are reliably transmitted by the lateral geniculate nuclei, which suggests the importance of maintaining temporal coupling of neural activities in processing the perception of global stimulus properties such as size and continuity of spatial features (Neuenschwander and Singer, 1996). The temporal coupling of peripheral neural activities between adjacent retinal ganglion cells is due to the presence of intercellular gap junctions (Roy et al., 2017). Studies of olfactory responses has also revealed temporal coupling of neuronal activities. Gilles Laurent and his colleagues observed that during an oscillatory response to odor in locusts, different neurons in the olfactory antennal lobe showed a higher probability of coincidental firing in a pair of neurons in some cycles but not in other cycles of the oscillatory response (Wehr and Laurent, 1996). Furthermore, neural oscillations play a pivotal role in various timing functions of the brain, including time perception (Buhusi and Meck, 2005; Gupta, 2014). In a recent study, recordings from the medial prefrontal cortex in monkeys, who produced different time-intervals using hand or eye movements, showed that the firing rate profiles were temporally scaled to match the produced intervals (Wang et al., 2018). This finding could be explained by the differences in the activation profiles of temporally-coupled subsets of neurons during the production of short and long intervals. Moreover, this study is consistent with the idea that the time course of the temporal coupling of neurons is responsible in part for the conscious time-interval production, while the scaling of the time course is correlated to the length of produced intervals.

## 3. Oscillations in the auditory brain stem as a temporal scale

**Figure 1:**
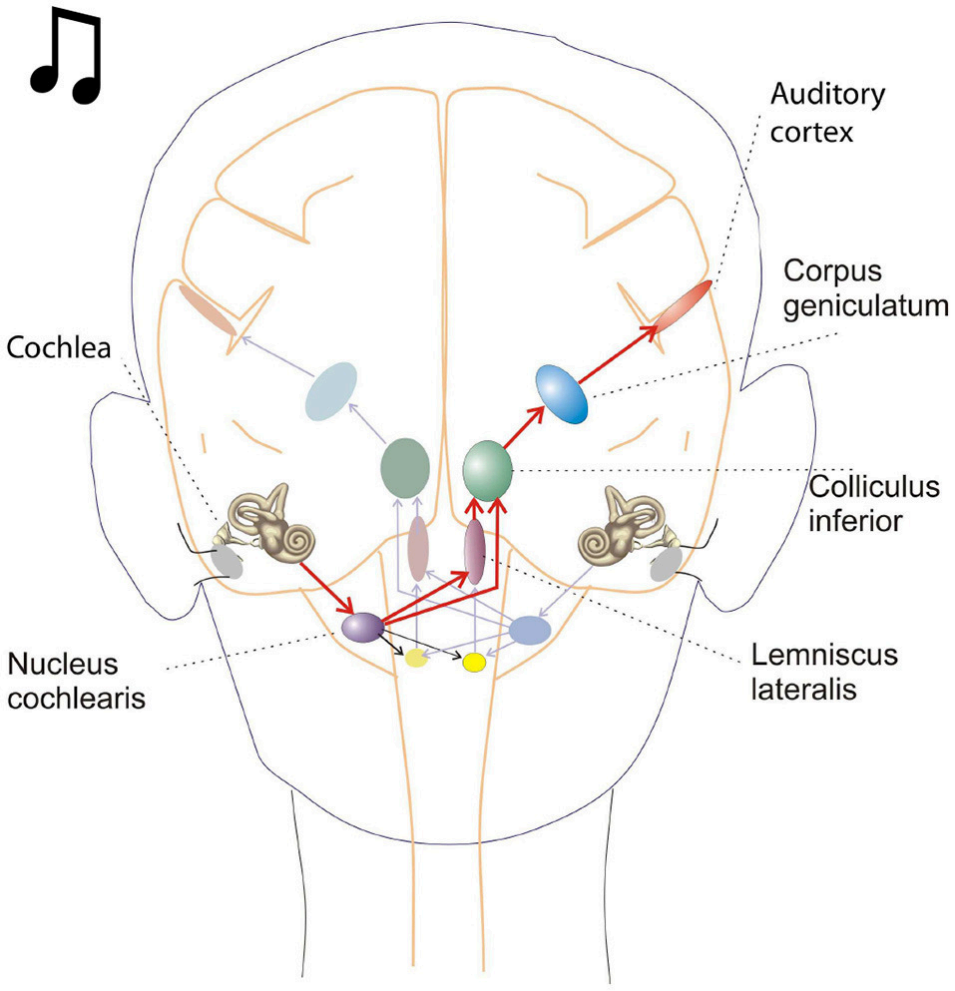
This schematic depicts key brain structures for processing auditory inputs. The cochlear nucleus in the brain stem is the initial processing center for auditory inputs and contains a variety of neurons capable of temporal processing. One such class of neurons, called chopper neurons, show a characteristic post stimulus time histogram, with ISIs (interspike intervals) which remain relatively constant and is unrelated to the stimulus frequency.

**Figure 2:**
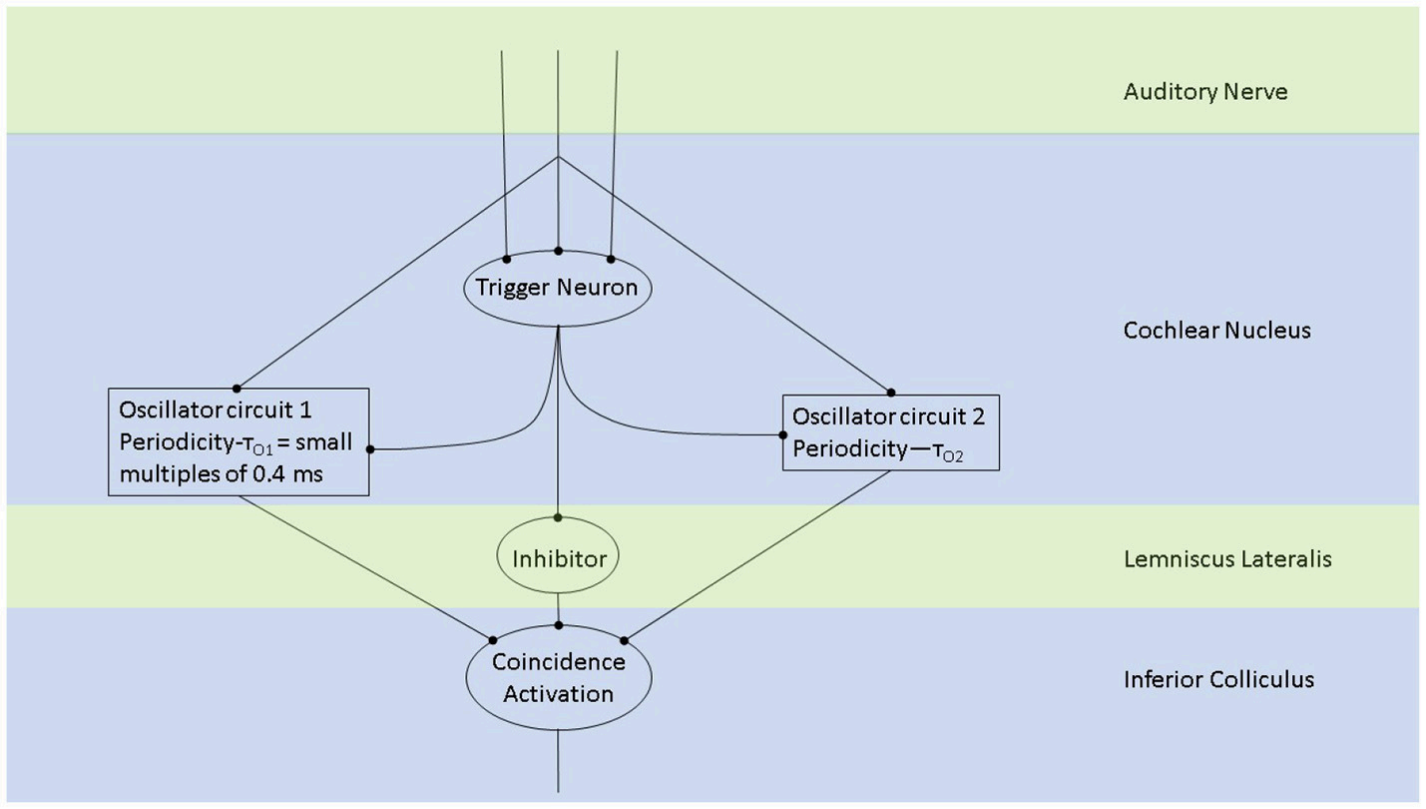
The periodicity analyzing neural model from Langner (2015) consists of two oscillators driven by the incoming auditory signal (Langner, 1981, 1983). Trigger neurons shown in this schematic are frequency-specific t-stellate cells/ chopper neurons, which give rise to tonic firing in response to phasic firing by the auditory nerve (Oertel et al., 2011). Successful periodicity analysis in the brain stem will result when coincidence activation takes place. Coincidence activation, projecting to the auditory cortex, can also account for sparse coding in the auditory cortex while there is tonic/phasic firing at the level of the brain stem and periphery.

**Figure 3:**
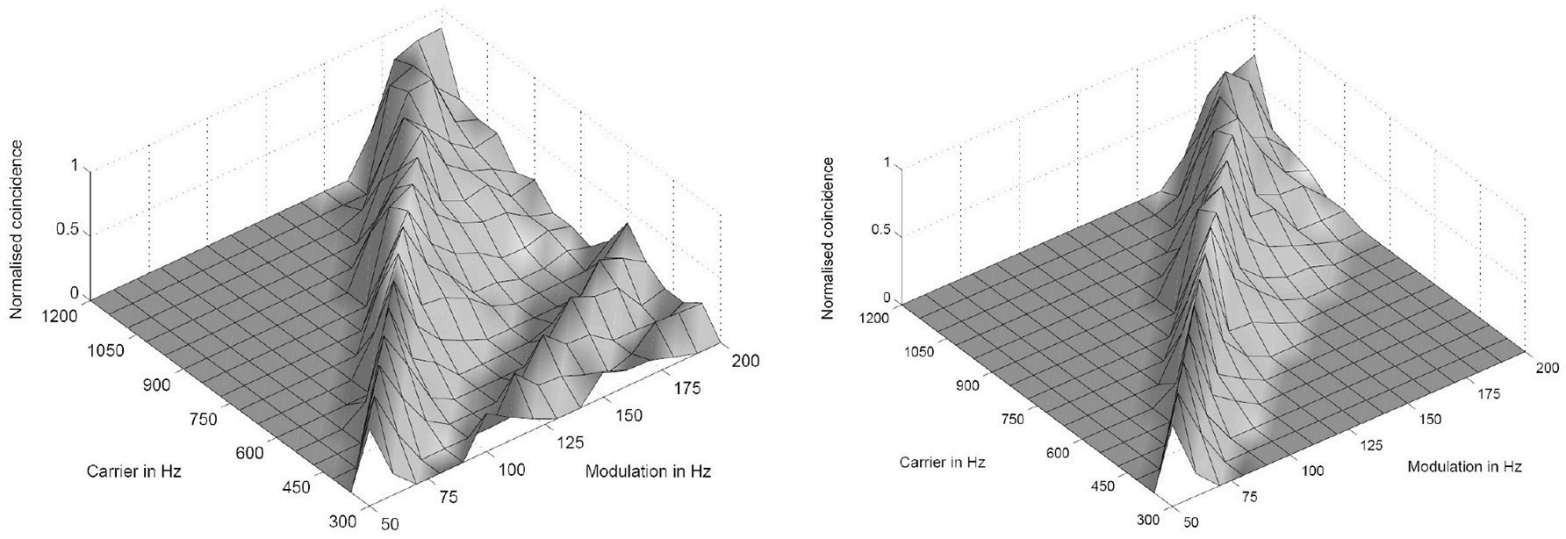
Simulation results of the periodicity model without **(Left)** and with **(Right)** an inhibitory connection. The response results from 16 periodicity models tuned to characteristic frequencies (CF) and best modulation frequencies (BMF) with the ratio 6:1 (CF/BMF). Stimuli are 256 combinations of 16 carrier and 16 modulation frequencies. The carrier frequency axes corresponds to the CF of one periodicity model (from Voutsas et al., 2005).

**Figure 4:**
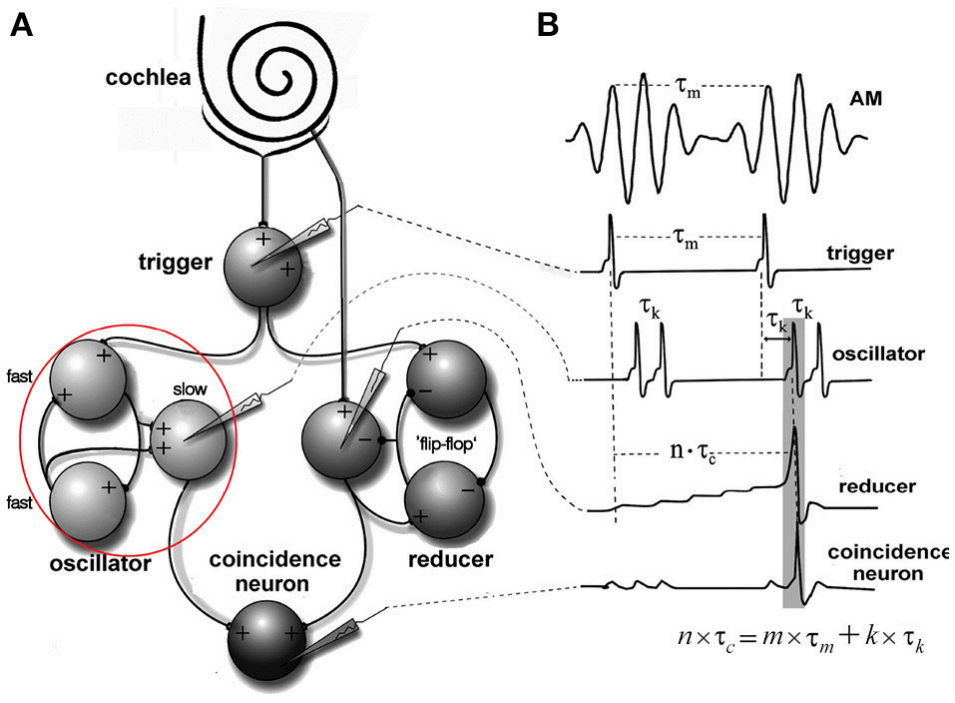
Periodicity model from Langner and Bahmer (Langner, 2015, chapter 9). **(A)** The model topology of the “oscillator” (red circle) contains fast and slow chopper neurons. **(B)** Corresponding “neuronal recordings.” In Figure 2, the “oscillator” corresponds to oscillator circuit 1 and the “reducer” to oscillator circuit 2.

Oscillations in the auditory pathways are observed in the cochlear nucleus and the inferior colliculus (Figure 1) among others. These oscillations are attributed to a class of neurons in the cochlear nucleus, called “chopper neurons” (see e.g., Blackburn and Sachs, 1989). Chopper neurons, which exhibit a unique response pattern, project to the inferior colliculus. They generate oscillations with a frequency, which is relatively independent of the changes of important stimulus parameters (Pfeiffer, 1966; Blackburn and Sachs, 1989; Wiegrebe and Winter, 2001; Winter et al., 2001). The interspike interval (ISI) of chopper neurons exhibit a distribution pattern in different species, which is centered at integer multiples of 0.4 ms (Langner and Schreiner, 1988; Bahmer and Langner, 2006a). In Mandarin, a tonal language wherein word meanings change with the pitch, periods, which are integer multiples of 0.4 ms can be found in statistically preferred tones (Langner, 2015). Recently, the temporal constant of 0.4 ms was found in electrophysiological recordings of the cochlear nucleus in human auditory brain stem implant patients (Bahmer et al., 2017). Chopper neurons play a key role in pitch perception (Langner, 1981; Hewitt et al., 1992; Wiegrebe and Winter, 2001). Incoming acoustical stimuli contain information about the pitch in their temporal modulation. Information about the temporal modulation is transferred via the auditory nerve to the ascending auditory pathways. The tuning of the auditory nerve fibers alone is not sufficient to explain the precision with which humans distinguish between pitch differences (just noticeable differences are about 0.2%, Fastl and Weinberger, 1981). Therefore, in addition to the coarse spectral analysis of the incoming signals in the cochlea, a subsequent temporal analysis is mandatory. Especially for absolute listeners, an inherent scale (neural oscillations in clock mechanism) could explain their outstanding ability to determine absolute pitch. Candidates producing such scales would be the chopper neurons in the cochlear nucleus of the brain stem. Chopper neurons have a significant role in the periodicity analyzing model introduced by Langner (1981, 1983) including the cochlear nucleus, inferior colliculus, and lemniscus lateralis. According to this model, a neuronal network including different types of oscillators (Figures 2, **4**) correlates features of the input signal to each other or correlates the features of the input signal to neuronal oscillations. In both modes, chopper neurons provide the temporal scale (Oscillator circuit 1, Figure 2). The function of the network is based upon the correlation of undelayed (oscillator circuit 1) and delayed neuronal responses (oscillator circuit 2) of the depicted neurons (Figure 2) to envelopes of amplitude modulated (AM) signals. These responses converge at neurons acting as coincidence detectors. Each modulation period of an AM signal activates the trigger neuron, which in turn activates a rapid oscillation (oscillator potential with a predefined frequency). Via parallel processing, the integrator neuron responds to the same cycle of the modulation frequency but with a longer delay which corresponds to the integration period from the integrator-like function. Moreover, the coincidence neuron will be activated, despite different delay intervals of the two previous units, provided that the integration period equals the period of the AM signal. A coincidence neuron will respond more often, when its inputs are synchronized, i.e., when the spikes of the oscillator and of the integrator converge synchronously. Thus, modulation periods (periodicity; τ_*m*_), m × τ_*m*_, with m = 1, 2, …, which activate the oscillations and drive the coincidence unit, can be computed according to the following linear equation:

(1) $$\begin{array}{r} m\times \tau_{m}=n\times \tau_{c}-k\times \tau_{k} \end{array}$$

where k, m, n are small integers. n × τ_*c*_ is the integration period, which consists of n carrier periods and after this interval the integrated input signal reaches a threshold. 1/τ_*c*_ is the carrier frequency of the AM signal, 1/τ_*k*_ the frequency of the auditory oscillations. Equation (1) will be referred here as coincidence equation. The parameter m takes into account the fact that coincidence neurons respond also to harmonics (m > 1) of the modulation frequency of the AM signal, which implicates ambiguity of IC neurons with respect to harmonically related signals. A solution to this problem is proposed by an input from the inhibitor (anatomically attributed to the lemniscus lateralis, a spiral structure). Because of the cochlear frequency analysis, neurons respond strongest at a characteristic frequency (CF). In addition to the CF, the coincidence neuron is tuned to a certain periodicity, i.e., a certain modulation frequency of an AM signal, also called the best modulation frequency (BMF). Therefore, different trigger, oscillator, integrator, and coincidence units are incorporated to explain the range of periodicity of AM signals (Langner, 2015). A detailed simulation of the periodicity analyzing model introduced by Langner (1981, 1983) can be found in Borst et al. (2004) and Voutsas et al. (2005). An example of the simulation results of the periodicity model with and without inhibition is depicted in Figure 3.

For the peripheral auditory system, ISIs of neural oscillations are argued to serve as a temporal scale (Bahmer and Langner, 2005). Absolute listeners may use this temporal scale for their outstanding ability to determine absolute pitch of the incoming tonal acoustic signals.

In a work presented here, we show that by simulating chopper neurons with various oscillation frequencies these neurons may serve a scale for a subsequent temporal analysis as for pitch determination. Furthermore, we hypothesize that microcircuits found in the auditory system which are dedicated to temporal analysis are ubiquitous in the brain for an operation in the temporal domain.

## 4. Neuronal modeling of oscillation in the auditory brain stem

The simulation of the oscillatory neuronal network in the auditory brain stem from Bahmer and Langner (2006b) are performed in Matlab 2006 (The MathWorks, Inc., Nattick) and NEURON (Hines and Carnevale, 1997). The differential equations are numerically realized by the Euler method in Matlab. Time steps of 25 μs are sufficient for the relevant time scales of about 0.1 ms. Signal, onset neuron, and chopper neurons are implemented as script-files, and auditory nerve fiber response is calculated within a mex-file in Matlab. Programs were executed on a PC with 2.0 GHz and 512 MB RAM.

The inner ear, inner stereociliary hair cells and auditory nerve fibers were modeled according to Hemmert et al. (2003). A wave-digital filter model describes the vibrations of the basilar membrane on the basis of the passive inner ear hydrodynamics; it consists of 125 mass-spring resonators that are connected by a coupling-mass (Strube, 1985; Zwicker, 1986). To simulate the outer hair cell function, the amplitude of the vibration of the basilar membrane is amplified and the traveling-wave along the basilar membrane is sharpened at the low values of the amplitude. This is performed by the second order resonators that are added at the outputs of the cochlear filter bank. The quality factors of the resonators are altered in all iteration steps depending on the displacement of each resonator. Four stages of the resonators are cascaded to achieve physiologically plausible amplification and filter shapes. Bundles of stereocilia of sensory hair cells are deflected by fluid motion from the movements of the basilar membrane (Mountain and Cody, 1999). When bundles of stereocilia are deflected, ion channels open and *K*^+^-ions diffuse into the sensory hair cells. The *K*^+^-ion diffusion depolarizes the inner hair cell membrane. Due to the depolarization, *Ca*^2+^-ions enter the cell through voltage activated Ca-channels. High *Ca*^2+^-concentration within the cell leads to the fusion of synaptic vesicles with the cell membrane (Moser and Beutner, 2000; Beutner et al., 2001). Specific quanta of neurotransmitter release are required to trigger the action potential at the postsynaptic membrane. Since there is a depletion of vesicles with release, spiking probability of the auditory nerve diminishes after a strong stimulus (adaptation). The model also includes a refractory period of about 1 ms (Carney, 1993). The generation of the action potential is a stochastic process due to the implemented random vesicle fusion. A single inner hair cell is connected to 20 synapses of the auditory nerve. Physiological and anatomical findings have led to the following simulation paradigm (Figure 4). (A) Two or three chopper neurons (fast) which are connected, can activate its subsequent neighbor, operate as a pace-maker, and project to other chopper neurons (slow) that have a longer refractory period. The fast neurons act as a pace-maker with a clock-rate of 0.4 ms. The slower chopper neurons which, due to longer refractory periods, skip short intervals while producing outputs at the long intervals, which are multiples of 0.4 ms. This reduces the number of the chopper neurons that are required to produce ISIs longer than 0.8 ms. (B) The first of two additional inputs are transmitted via five synapses from the auditory nerve fibers (Ferragamo et al., 1998a). (C) The additional input comes from the onset neuron and activates only one of the chopper neurons in the circuit. The onset neuron (trigger) receives its broadband input from the auditory nerve and excites one chopper neuron (fast). Inputs from the auditory nerve depolarize the membrane of the chopper neurons. This change in the membrane voltage enables chopping but does not initiate it. The reason is that the weights of auditory nerve synapses are adjusted in such a way that the auditory nerve input alone cannot drive the membrane voltage to the threshold. Instead, the chopping is initialized by a spike from the trigger/onset neuron. The onset neuron is a simplified version of the model that was proposed by Rothman and Manis (2003) and is based on Hodgkin-Huxley (HH) equations. The model consists of a sodium (*I*_*Na*_), a low-threshold potassium (*I*_*LTK*_), an excitatory synaptic (*I*_*E*_) and a leakage (*I*_*lk*_) current. The low threshold of the potassium channel opening is responsible for the onset neuron behavior (Rothman and Manis, 2003). Simulation parameters of the adapted HH-like onset neuron can be found in Bahmer and Langner (2006b). Chopper neurons are modeled as leaky integrate-and-fire neurons with synapses (Bleeck, 2000). The synapses are modeled as follows. The action potential in the presynaptic neuron leads to the fusion of vesicles, discharging neurotransmitters into the synaptic cleft. The emission of vesicles is simulated by use of a look-up table. The neurotransmitter molecules traveling in the cleft to the postsynaptic neuron is modeled by diffusion. The decay of neurotransmitter effect is simulated by a leaky integrator. The probability of open channels for certain ions increases as the concentration of neurotransmitter in the synaptic cleft becomes higher. Various ions produce either excitatory or inhibitory postsynaptic currents. A hyperbolic tangent function controls the channel conductance. A time delay with adjustable jitter (parameters: mean and standard deviation) that stands for the overall neurotransmitter diffusion time was integrated in the simulation. Details like the neuron and synapse model equations and simulation parameters can be found in Bahmer and Langner (2006b). The simulation of chopper neuron soma activity is based on a leaky integrate-and-fire model. The incoming postsynaptic currents from the synaptic inputs are integrated and build up a postsynaptic potential while a leakage current diminishes the input. When the potential reaches a predefined threshold, a spike is elicited, and the membrane potential is reset. The absolute and relative refractory period (exponentially decreasing) ensures that the spike generation is suppressed or needs a stronger input, respectively, for a given period of time. The time constant of the fast chopper neurons in the simulation is set to 0.8 ms to ensure a fast chopping; whereas the time constants of the slow chopper neurons is set to higher values according to their low chopping frequencies. The summed weight of the synapses of the nerve is on average eight times lower in the simulations than the weights of the synapses of the chopper and onset neuron. Excitatory postsynaptic potentials lead to the subthreshold depolarization of the membrane to enable chopping. This weak auditory nerve input does not mean that the overall response of the chopper neuron is low because the input from the network also contributes to the response. As an alternative to the leaky integrate-and-fire chopper neuron model described in the previous section, the HH-like chopper model of Rothman and Manis (2003) for the simulation environment NEURON was simulated (Bahmer and Langner, 2010). According to the results, the model has the disadvantage that it cannot reproduce *in vivo* data of subpopulations of chopper neurons showing small ISIs (e.g., 1.4 ms, Young et al., 1988). Moreover, the dynamic range of the spike rate of real chopper neurons is about 200–300 spikes/s in average (Frisina et al., 1990). If this physiologically dynamic range is applied to the simulation, the corresponding ISIs in the simulation span a range of about 5–23 ms, whereas *in vivo* values of ISIs differ much less with varying levels (e.g., Frisina et al., 1990). Therefore, the model was adapted by means of genetic algorithms (Bahmer and Langner, 2010) which resulted in cell parameters in a physiologically plausible range. For the simulation of the modified model, the currents are varied in NEURON and the corresponding voltage responses are saved. The voltage responses were then analyzed in Matlab and the ISIs were plotted versus the input strength. For the neuronal modeling II, the auditory nerve input is modeled as a signal step and the onset neuron is modeled as a single-spike generator.

## 5. Simulation of a small network of fast pacemaker neurons in the auditory system

**Figure 5:**
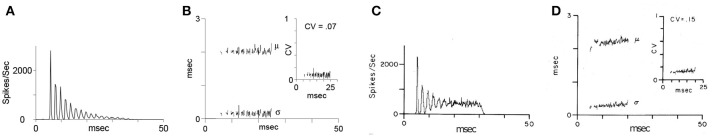
“Oscillator” response from Figure 4: Simulated chopper neuron **(A,B)** and recording of a sustained chopper neuron **(C,D)** in the CN of the cat (Blackburn and Sachs, 1989). **(A,C)** Peri-stimulus time histogram (PSTH) response to 500 stimuli (bin width: 0.3 ms). **(B,D)** Regularity analysis: mean (μ), standard deviation (σ), and coefficient of variation (CV) of interspike intervals. For simulated and *in vivo* responses, stimuli were short tone bursts (25 ms, 1.6 ms rise and fall time) with frequency at the CF of the chopper neuron (2.89 kHz), 30 dB above threshold.

Blackburn and Sachs (1989) classified (anterior ventral) cochlear nucleus neurons using regularity analysis of ISIs. Important parameters of this analysis were mean and standard deviation. The coefficient of variation value (CV, ratio: standard deviation to the mean of ISIs) enables a comparison of different units of chopper neurons and different stimulus levels. The CV is computed as a function of time. Sustained chopper neurons are a subtype of chopper neurons and classified by a small CV, indicating their highly regular ISIs. Figure 5 shows the simulation results of the multi-oscillator and physiological data of a sustained chopper neuron in the CN (Bahmer, 2007). Firing rate and ratio of peak heights match their known physiological properties. The data obtained after the simulation, such as firing rate, number of peaks, and ratio of peak heights are similar to electrophysiological data. Even the regularity analysis could be matched to i data. In this simulation, a jitter (standard deviation 0.26 ms) is added to the synaptic delay of the interconnections of the fast chopper neurons.

## 6. Simulation of a small network of slow pacemaker neurons in the auditory system

**Figure 6:**
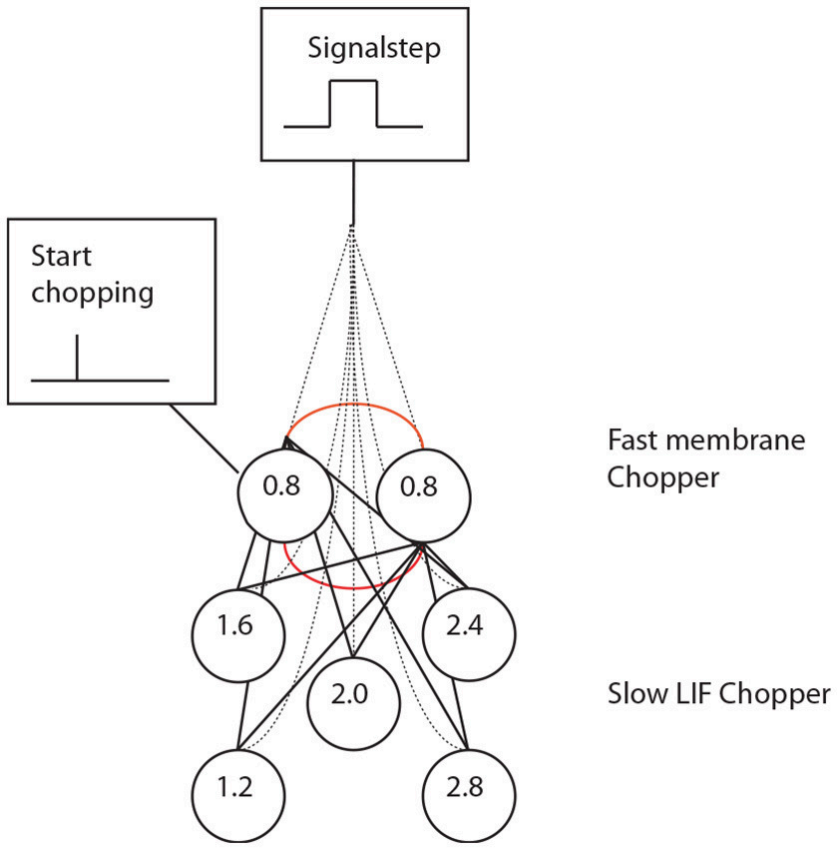
A set of chopper neurons provides time intervals that are multiples of 0.4 ms by reducing a high-frequency input from a pacemaker micro-circuit.

**Figure 7:**
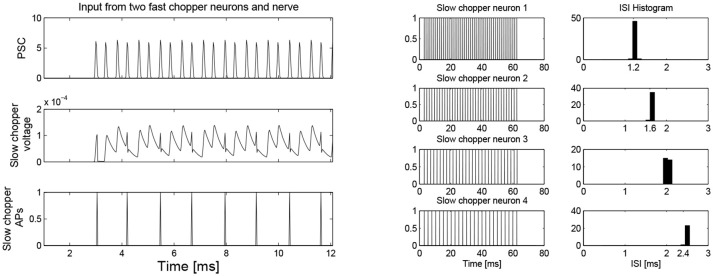
**(Left)** Simulation of slow chopper neuron that receives input from two fast chopper neurons and the auditory nerve. **(Right)** Various integer multiples of 0.4 ms can be provided by a small number of chopper neurons, which receive an input from the same pacemaker.

Simulation with the adapted model (Figure 6, see also Bahmer, 2007) shows oscillations with ISIs of 0.8 ms duration. Two of these adapted neurons can mutually excite each other and act as pacemaker. This pacemaker projects to other chopper neurons that have slower time constants and, therefore, skip a certain number of spikes. Nevertheless, the skipping results in ISIs with are integer multiples of 0.4 ms (Figure 6). In the simulation, the post synaptic current (Figure 7 left, PSC) drives the membrane voltage of the slow chopper neuron to the threshold but due to the refractory period several supra-threshold inputs are skipped. Only action potentials at every third supra-threshold input are elicited. Thus, action potentials are only elicited at every third supra-threshold input (ISI: 1.2 ms). For a set of slow chopper neurons, action potentials with various ISI (integer multiples of 0.4 ms) are generated which depends on the refractory period. Note that the refractory period is not necessarily an integer multiple of 0.4 ms, but is a continuous variable; however, ISIs are integer multiples of 0.4 ms, corresponding to the periodic inputs from the fast chopper neurons.

As it can be noted from equation 1, the solution for the correlation of the integration period of the carrier, the modulation frequency, and frequency of auditory oscillations is constrained by integer values of m, k and n. The integer values of m, k and n would represent the number of oscillations, which are reached in respective circuits before integration, a correlate of perception occurs. In fact, as discussed later, circuit patterns found in the auditory system for an effective analysis of high temporal informational content can be found throughout the entire brain (Oertel and Young, 2004; Langner, 2015). We review literature, which shows that many microcircuits, which employ coincidence detection mechanism to temporally couple neural events, are found across the brain.

## 7. Inhibition of the self-exciting oscillator microcircuit in the auditory brain stem

The simulation of a cluster of chopper neurons shows that oscillations with precise ISIs can be generated with the help of a few neurons. Two or three interconnected fast chopper neurons act as a pacemaker with a smallest temporal resolution of 0.4 ms projecting to the slow chopper neurons. The slow neurons can skip supra-threshold inputs and generate outputs at longer ISIs. In physiological measurement ISIs span a wide range of durations (Young et al., 1988). In the simulation from Bahmer and Langner (2006b), chopper neurons can excite each other as observed in T-stellate cells (Ferragamo et al., 1998b). T-stellate cells also receive an inhibitory input from D-stellate cells. This input, in the presence of the input from the auditory nerve, can inhibit the self-excitation of the network. In the simulation from Bahmer and Langner (2006b), the offset at the end of the input from the auditory nerve was sufficient to stop the excitation of the network. In a future version, the input from D-stellate cells shall be included as excitation must be balanced by inhibition especially if the network contains more interconnected chopper neurons. Furthermore, a combination of inhibitory and excitatory inputs enhances the signal detection and provides means of gain control by reducing noise by inhibition (Caspary et al., 1994; Josephson and Morest, 1998).

For the fast chopper neurons, this input enables chopping; it is a condition for starting and stopping the chopper neurons and is necessary in a self-exciting network (Bahmer and Langner, 2007). But, in the context of the current model, this does not seem to be necessary for the slow chopper neurons because this functional role is substituted by the projection of the fast chopper neurons. On the other hand, if an additional inhibition of chopper neurons is included (see above: functional role of inhibition of D-stellate cells) this input again seems reasonable. However, if the inhibition is strong enough to mute the circuit, the onset neuron would not activate the chopper neurons. With the help of the excitatory inputs from the auditory nerve, the inhibition is balanced, and the onset neuron is able to activate the chopper neurons. Moreover, the integration of inhibition in this model can plausibly enhance dynamic processing (Eguia et al., 2010).

## 8. Transformation of incoming auditory information into a sparse code

Psychoacoustical studies in the past have indicated that the perception of speech is not adequately accounted by place frequency mechanisms (Rosen, 1992). The temporal information represented in sounds is also important in the perception of speech (Rosen, 1992). Therefore, it is noteworthy that a recent theoretical work and a growing number of experimental studies indicate that time-dimension is an integral part of information processing underlying various perceptual functions (Gupta, 2014; Gupta and Chen, 2016). Most natural sounds are modulated in amplitude (Joris et al., 2004; Eguia et al., 2010), and, thus, they are represented by two frequencies: a fast frequency, which represents fine oscillations of sound waves and a slow frequency of the amplitude modulation. The oscillations of both frequencies, forming the structure of AM signals of natural sounds processed by cochlea, help to represent physical time-dimension (Gupta, 2014). The spike structure of the AM signals is phase locked to the movements of inner hair cells, which directly results from the pressure changes produced by amplitude-modulated sound waves. Thus, oscillatory structure of AM signals inputs temporal information into neural circuits when they are processed by trigger neurons (Figure 2). Moreover, this is consistent with the discussion of equation 1, based on the periodicity analyzing model (Langner, 1981, 1983) which suggests that both the carrier frequency of sounds as well as its modulation frequency are responsible for the integration underlying perception. The coincidence detection (Figure 2), responsible for integration would result in a sparse code (Harris et al., 2011), which would be processed in the cortical auditory areas to create the perception of sound.

## 9. Coincidence detection via distributed microcircuits is a key mechanism for conscious brain functions

**Figure 8:**
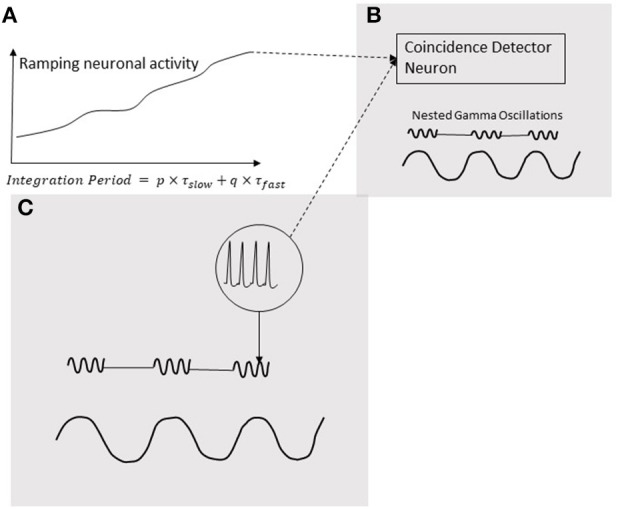
Coincidence detection of three events. Two of these events are gamma oscillations nested in the excitation phase of a low-frequency oscillation **(C)**; the third event is a ramping activity of a neuron **(A)**. The output of the neuron ramping activity stimulates the neuron **(B)** in the brain area synchronized with the nested oscillation. The neuron in **(B)** will be stimulated when ramping activity reaches a threshold coinciding with the nested gamma oscillations. The time-period from the start the ramping activity, called Integration Period, will encode the timing of the action.

Neural oscillations are hypothesized to play a pivotal role in decoding the temporal information in ramping neuronal activities (Gupta, 2014) that are commonly observed in the cortex (Leon and Shadlen, 2003; Durstewitz, 2004; Lebedev et al., 2008; Schneider and Ghose, 2012; Narayanan, 2016). As discussed in the Introduction, temporal coupling of neural events is important for various cognitive functions of the brain. Moreover, the temporal coupling can be realized by coincidental activation of neural circuits. Furthermore, our models support the role of coincidence detection in the analysis of temporal information in auditory signals. Coincidence detection would play a key role in generating the information that produces a consciously timed behavior. According to the schematic in Figure 8, this information is processed when coincidence detector neuron is stimulated by both, excitatory presynaptic terminals controlled by gamma oscillations (Fries, 2015) as well an increasing excitatory input coming from a ramping neuronal activity. In this mechanism, the ramping activity of neurons resembles an integrator and the oscillators periodicity determine the limit of integration. A coincidence detection model (Figure 8), based on the periodicity analyzing model for auditory signals proposed by Langner (2015), can provide a basis for decoding the information coded by the pattern of ramping activity. As argued by Gupta and Chen (2016), action and perception are temporally coupled by hierarchical neural oscillations. Consistent with this, a coincidence detection of three events is depicted in Figure 8. Two of these events are fast-(gamma) oscillations nested in the excitation phase of a slow-oscillation (Figure 8C). The third event is the ramping activity of a neuron (Figure 8A). The output of the neuron with the ramping activity stimulates the neuron (Figure 8B) in the brain area synchronized with the nested oscillation. The neuron in (Figure 8B) will be stimulated when ramping activity reaches the threshold, coinciding with the nested gamma oscillations. The time-period from the start of the ramping activity, called Integration Period, will encode the timing of the action.

Cross-frequency coupling allows discrete packets of high (gamma band) frequency oscillations to be formed across larger areas of the brain synchronized by low (alpha and beta bands) frequency oscillations (Buzsáki and Watson, 2012; Gupta and Chen, 2016). The excitatory phase of neural oscillations can increase the probability of coincidental firing of neurons leading to information processing via discrete circuits in a network. Furthermore, according to a leading modern theory of perception, predictive coding, there is an interaction between feedforward and feedback information (Friston, 2008). Cross-frequency coupling would lead to integration by climbing neuronal activities in the cortex during interaction between feedforward and feedback circuits. Experimental evidence and theoretical considerations, reviewed earlier (Bastos et al., 2012), suggest that feedforward connections, predominantly present in the superficial layers of the cortex, use higher frequency oscillation (gamma range), compared to alpha or beta frequency used by feedback connections in the deep cortical layers.

(2) $$Integration\;Period=p\times \tau_{slow}+q\times \tau_{fast}$$

τ_*slow*_ and τ_*fast*_ are periodicities of slow- and fast-oscillations, and p and q are integers. Ramping activities could also play an important role in the analysis of multiple inputs that underlies a decision process. Single cell recording from layer 5 in the primary motor cortex of rats had shown that there is a strong modulation of specific neuronal activity when there are unfamiliar movements, such as the right or left movements (Cohen and Nicolelis, 2004), which is a suggestive of a decision process. Moreover, the neurons in the cortical layer 5 send axons to the thalamus, basal nuclei, brain stem as well as the spinal cord to control motor movements (Crossmann and Neary, 2010). Since the primary motor cortex receives inputs from the prefrontal cortex and different sensory areas (Borra and Luppino, 2017; Kheradmand and Winnick, 2017), ramping activity may result from a variable balance of inputs from many of these areas, which would be the basis for the decision process.

## 10. Anatomical substrates for canonical microcircuits for temporal processing in the brain

**Figure 9:**
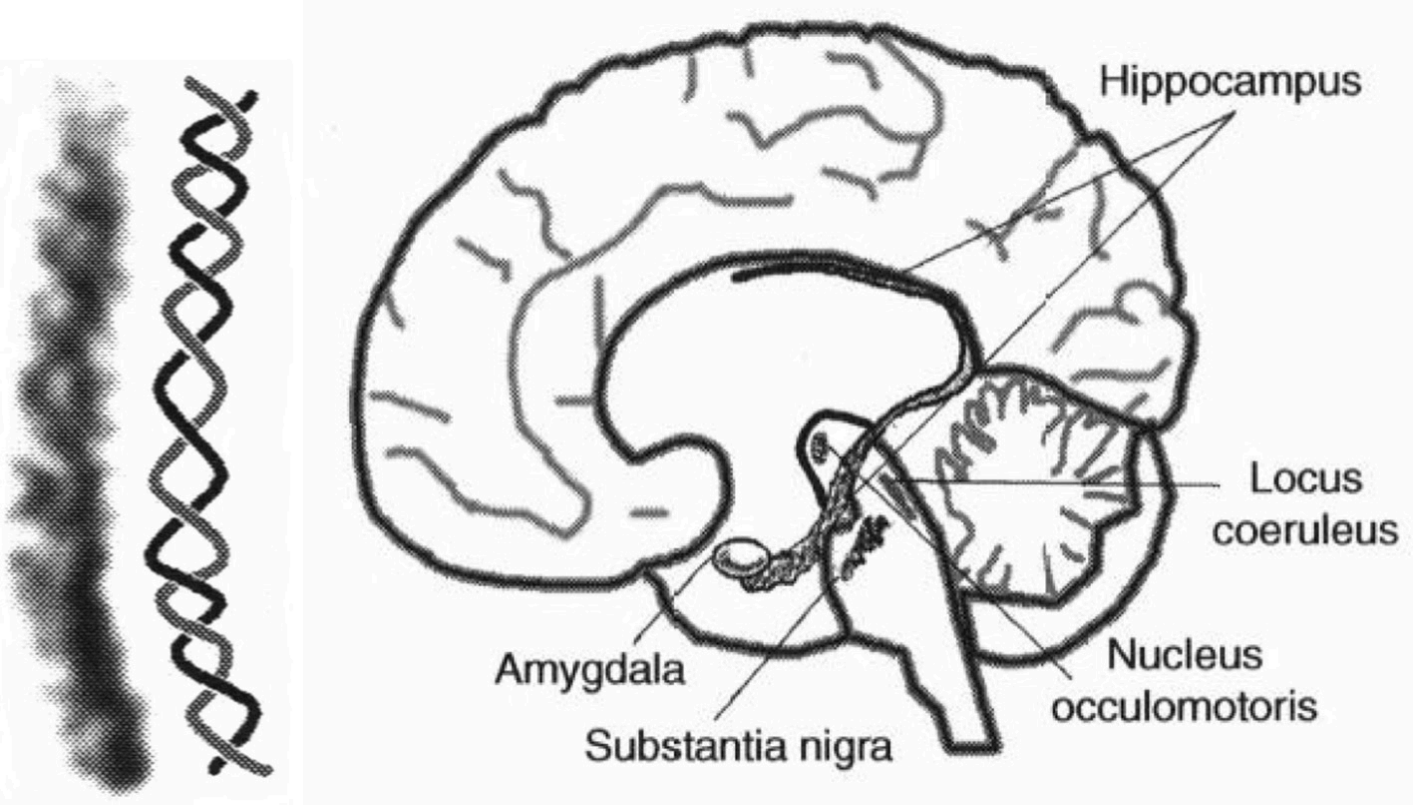
**(Left)** Example for a helical-like structure in the hippocampus. **(Right)** Helical-like structures can be seen in various locations in the brain, such as the basal ganglia (substantia nigra), reticular formation (locus coeruleus), and limbic system (hippocampus, amygdala), both reproduced from Langner (2015) with permission.

The auditory system has evolved by adapting its internal functional structures for a fast processing of incoming signals. As outlined in the Introduction, a periodicity analysis of incoming signals can be accomplished by simple neuronal elements (Langner, 1981). These elements resemble components like integrators, differentiators, and temporal coincidence detectors. Even the occurrence of harmonics in the periodicity analysis—the unwanted side effect of a correlation analysis see Figure 3—is suppressed by a helical structure located in the lemnisculs lateralis (Ochse, 2004; Voutsas et al., 2005; Langner, 2015). Note that oscillations are ubiquitous in the brain as outlined in the Introduction. However, in contrast to their specific functional role as a temporal scale in the auditory brain stem, they are rather seen as an epiphenomenon in other brain areas, that is, no distinct meaning can be generally attributed to a certain oscillation frequency. Nevertheless, oscillations are a power tool for communication between neuronal networks (Gray and Singer, 1989; Gray, 1994; Fries, 2015). Given that temporal neuronal processing is enhanced by oscillations, it is not surprising to find similar canonical microcircuits in the brain (e.g., the cerebellum-like circuit pattern found in the dorsal cochlear nucleus and pallidum, see Oertel and Young, 2004). There are several parts of the brain that contain helical-like structures after reconstructing from sections, and resolved at the level of cells [Figure 9, ventral part of the lemniscus lateralis, locus coeruleus, oculomotor nuclei, amygdala, hippocampus (cornu ammonis 3), and pars compacta and reticulata of the substantia nigra, Langner (2015)]. These structures provide plausible anatomical solutions for processing hierarchical oscillations as there could be at least two gradients of frequencies in ensembles of neurons: one from periphery to the center and the other between several turns of the helix (Langner, 2015).

## 11. Oscillations as a target for brain-computer-interfaces

It has always been a vision to interface the brain with a computer to control brain functions. In the auditory system, computer-brain interfaces have already become reality with the development of cochlea, brain stem, and midbrain implants. Cochlea implants stimulate the auditory nerve in the cochlea with electrical impulses, brain stem implants are located in the cochlear nucleus, midbrain implants in the inferior colliculus. These implants are still undergoing further improvements through research, and understanding the role of the oscillations in the cochlear nucleus may be the key to further improvements. In addition, a resonance phenomenon may help to locate target structures for auditory brain stem implants. Ramsden et al. (2016) have postulated the existence of chopper neurons with a preference for certain oscillations periods (Bahmer and Langner, 2006a,b) as a target for electrical stimulation. Based on the idea of targeting certain neuronal networks, strategies have been proposed in electrical stimulation of neuronal networks for cochlear implants, auditory brain stem implants, auditory mid brain implants, as well as for deep brain stimulation (Bahmer et al., 2009; Bahmer, 2016, 2017; Bahmer and Schleich, 2016). These stimulation strategies and alternative pulse shapes (Bahmer et al., 2010; Bahmer and Baumann, 2016) may also be useful for the deep brain stimulation in psychiatric diseases (Buzsáki and Watson, 2012).

## 12. Oscillations underlying auditory steady state responses: impact on schizophrenia and depression

Studies have shown that the perception of sound waves is associated with an increased inter-hemispheric interaction via synchronization long-range gamma bands (Steinmann et al., 2014). Gamma oscillations could play a key role during the long-distance synchronization of local circuits in this inter-hemispheric interaction (Buzsáki and Watson, 2012; Fries, 2015). In each gamma cycle, there is a state of excitation, lasting 3 ms, which triggers an inhibition, lasting for the remainder of the gamma cycle (Fries, 2015). The precision of the 3 ms excitation in the gamma cycle may help to temporally align neural events via long-range gamma band synchronization (Steinmann et al., 2014) in circuits, subserving the perception of sound waves in two hemispheres. Thus, the perception of sounds could be causally related with the temporal coupling in cortical areas, which would result from the coincidence detection events, similar to the processing of auditory signals in the brain stem.

In schizophrenia, which is characterized by the impairment of the perceptual functions, patients often suffer from hallucinations. Thus, it is not surprising that a meta-analytic study finds that in schizophrenia, there is a reduction in the power as well as phase locking values of the 40 Hz gamma-range auditory steady state responses (ASSR) (Thuné et al., 2016). This is consistent with a reduction in the temporal coupling of neural activities, processing sound stimuli in schizophrenia, which would be responsible for the impairments in sound perception, contributing to auditory hallucinations. In addition, ASSR is also affected in bipolar disorder (Rass et al., 2010).

Depression is the most prevalent psychiatric disease (a roughly 20% lifetime incidence in Western populations) and the third largest amongst all illnesses in the world (Mathers et al., 2008). Abnormal differences in oscillations after auditory stimulation have been found between depressed patients versus controls (Iosifescu, 2011). Treatment options are restricted, and the medication success is often based on trial-and-error and a relevant question is whether a particular measure can predict the outcome of the treatment (Buzsáki and Watson, 2012). Interestingly, the loudness-dependence of auditory evoked potentials, can determine the responsiveness to serotonergic versus non-serotonergic antidepressants (Hegerl and Juckel, 1993; Iosifescu, 2011).

## 13. Conclusion

In this review, we discuss how temporal information in auditory signals can be accurately analyzed by means of the oscillating activity of chopper neurons in the brain stem. This analysis involves the activation of coincidence neurons, which detects the temporal coupling between the discharges by circuits of chopper neurons with a regular firing pattern, and the integrator neurons with a ramping activity pattern (Figure 4), which would project to the cortex as a sparse code. Moreover, neurons with ramping activity, resembling the integrator neurons, are commonly found across the cortex. Mechanisms involving coincidence detection neurons, modulated by nested gamma oscillations may contribute to the information processing that decodes the activity of ramping neurons (Figure 8). Additionally, it should be noted that the coincident activation only detects spatiotemporal convergence of neural events; however, primary triggering events may be few milliseconds apart (Fries, 2015). Coincidence detection of neural events, is also likely to form the basis of a variety of perceptions, such as sensations of smell, sound, even the spatial perception of visual objects. As noted above, the impairments of temporal coupling could also contribute partly to the defects of conscious functions in schizophrenia, bipolar disorder, depression, just to name a few. Accordingly, the future investigations of the temporal coupling in the brain may help us develop new treatments of some of the most socially devastating ailments.

## Author contributions

AB and DG conception, revising, final approval, approving of publication and accountable for all aspects of the work. AB simulation, data analysis.

### Conflict of interest statement

The authors declare that the research was conducted in the absence of any commercial or financial relationships that could be construed as a potential conflict of interest.
